# Blood analytes of clinically normal and diseased neonatal and weaned farmed white-tailed deer (*Odocoileus virginianus*) fawns

**DOI:** 10.1080/01652176.2023.2249072

**Published:** 2023-08-24

**Authors:** Allison R. Cauvin, Samantha M. Wisely, Benjamin Baiser, Rebecca M. Peters, Katherine A. Sayler, Jeremy P. Orange, Jason K. Blackburn, Nicole I. Stacy

**Affiliations:** a Department of Wildlife Ecology and Conservation, University of Florida, Gainesville, FL, USA; b Department of Geography, University of Florida, Gainesville, FL, USA; c Department of Comparative, Diagnostic, & Population Medicine, College of Veterinary Medicine, University of Florida, Gainesville, FL, USA

**Keywords:** Biological variation, intrinsic factors, reference intervals, weanlings

## Abstract

Recent research focused on farmed deer has exposed many knowledge gaps regarding health assessment protocols for white-tailed deer (WTD). The objectives of this study were to establish de novo blood analyte reference intervals for farmed WTD fawns at birth (1–2 days of age; *n* = 84) and again at weaning (76–125 days of age; *n* = 28), to compare data at birth and at weaning to understand how these analytes are affected by the intrinsic factors age and sex in clinically normal WTD fawns, and to compare between clinically normal and sick WTD weanlings (respiratory disease *n* = 12; orbivirus-infected *n* = 6). Reference intervals were established for WTD fawns at birth and weaning. Female WTD neonates had significantly higher red blood cell counts, hematocrit, and hemoglobin compared to males. Most blood analytes were significantly different in clinically normal WTD neonates compared to weanlings, suggesting an effect of age. The observed sex- and age-related variations in WTD highlight the need to establish reference intervals that account for intrinsic factors. The comparison of clinically normal and sick WTD weanlings in this study identified higher MCHC and absolute monocytes in sick weanlings but these findings were presumably not biologically relevant given the small sample size for sick fawns. While the reference interval data presented herein will be useful for the veterinary care of WTD fawns at critical time periods in a high-density farm setting, this study also demonstrates the need to identify more sensitive and specific biomarkers for the assessment of health status in farmed WTD with specific underlying diseases.

## Introduction

Cervid farming represents a viable and important industry in rural America with an estimated total economic impact of $3.0 billion nationally (Anderson et al. 2007). Fawns represent the basis of production for cervid farmers and the future genetics of the industry; therefore, a high survival rate is essential for operational success. Farmed white-tailed deer (WTD) fawns are disproportionately affected by acute bacterial infections, particularly respiratory tract disease caused by *Fusobacterium* spp (Brooks and Jayarao 2008; Brooks et al. 2014). and *Trueperella pyogenes* (formerly *Arcanobacterium pyogenes*), and enterocolitis due to *Clostridium perfringens* and *Escherichia coli* (Hattel et al. 2016). In addition to bacterial infections, significant causes of mortality in fawns include epizootic hemorrhagic disease virus (EHDV) and bluetongue virus (BTV) in areas where these are endemic (Howerth et al. 2000). WTD may be exposed several times to EHDV and BTV, and fawns have passive immunity due to the presence of maternal antibodies (Ruder et al. 2015). Periodic outbreaks of these *Culicoides*-borne viruses occur during late summer to early autumn when fawns begin to lose their protective maternal antibodies (Howerth et al. 2000; Gaydos et al. 2002). Due to the acute onset and etiologic diversity of these frequently encountered diseases in fawns, rapid and effective diagnostics are necessary for early intervention and development of monitoring and treatment strategies.

While several diagnostic tools are used to assess WTD health, none are without drawbacks. For instance, nucleic acid-based assays typically depend on polymerase chain reaction (PCR) detection of disease-causing agents, including the detection of viral RNA (vRNA) from EHDV and BTV. While accurate, these methods can be expensive, time-consuming, and rely on the extraction of rapidly degrading genetic material by experienced laboratory personnel. Antibody assays, such as virus neutralization tests (Stallknecht et al. 1995) or commercially available enzyme-linked immunosorbent assays (ELISAs), while generally useful for determining antigenic exposure, cannot differentiate between current and past infections. Therefore, the application of other diagnostic modalities for common conditions and diseases in farmed WTD is warranted.

Blood analyses, such as complete blood count (CBC) and serum biochemistry are some of the most cost-effective and readily available methods of health screening and for diagnosis of the presence of underlying disease in any species (Harvey 2012). Additionally, protein electrophoresis and the acute phase protein serum amyloid A have been shown to have potential utility for the health assessment of WTD (Cray et al. 2019). Blood analysis for hematology and serum chemistry of WTD fawns has been the subject of multiple investigations given the importance of WTD as a game species. Hematological analytes of captive and free-ranging WTD fawns are useful indicators of general health and nutritional status (DelGiudice et al. 1987; Rawson et al. 1992; Sams et al. 1996). Intrinsic factors, such as age and sex have been found to influence hematological data in WTD, likely due to metabolic and hormonal shifts (Seal and Erickson 1969; Tumbleson et al. 1970). Experimental infections in WTD with EHDV-2 and BTV-10 have been previously associated with changes in blood analytes, specifically marked lymphopenia of <1000 cells/µl in severely affected animals, indicating that hematological data may be useful in differentiating deer with active orbiviral infections (Quist et al. 1997).

Many of the previously published studies included farmed deer at lower animal densities, have not specifically evaluated the crucial neonatal or weaning time periods in their analyses, presented a limited number of blood analytes, or have not been generated according to American Society for Veterinary Clinical Pathology (ASVCP) guidelines as outlined in Friedrichs et al. (2012). Since it is generally understood that hematology and serum biochemistry data can be influenced by many factors, such as age, sex, geographic location, nutritional status, and stress (DelGiudice et al. 1987; Rawson et al. 1992; Sams et al. 1996), these previous studies may not reflect the unique environmental and husbandry conditions that WTD fawns encounter in a farmed setting in Florida. Therefore, this study sought to focus on these knowledge gaps. The objectives of this study were (1) to establish de novo hematological and serum biochemical reference intervals for farmed WTD fawns at birth (1–2 days of age; *n* = 84) and at weaning (76–125 days of age; *n* = 28), (2) to compare blood analyte data at birth and at weaning to understand how these analytes are affected by the intrinsic factors age and sex in clinically normal WTD fawns, and (3) to compare these data between clinically normal and sick WTD weanlings (respiratory disease *n* = 12; orbivirus-infected *n* = 6). This study will support improvements in veterinary care of WTD fawns at critical time periods, specifically when deer farmers are routinely handling their stock at birth and weaning.

## Materials and methods

### Ethics statement

This study was approved by the University of Florida’s Institutional Animal Care and Use Committee (IACUC) as study #201508838.

### Study population

Blood samples from neonatal (*n* = 111) and weaned (*n* = 49) WTD fawns were collected from a cervid farm located in Gadsden County, Florida, between May 2016 and September 2017. Pregnant dogs were fed commercially available, high-protein feed (Record Rack–Sportsman’s Choice, Cargill, Minneapolis, MN, USA). Additionally, the pens were seeded with improved forage of Bahia and Florida native grasses. Farmed animals were kept in pens at high densities (∼1200 animals/km^2^), as opposed to the 8 animals/km^2^ wild population density estimates reported in northwest Florida (Cauvin et al. 2020). At the time of birth, neonates were provided with probiotic fawn paste (C&E Wildlife Products, Laredo, TX, USA), as well as Clostridium C&D antitoxin (Clostratox BCD, Novartis, Basel, Switzerland). All neonatal fawns included in the study were classified as clinically normal upon handling based on visual examination (e.g. bright, alert, absence of external injuries, absence of any overt clinical abnormalities) and were tested for orbivirus infection by RT-qPCR as described below. One animal was excluded from the study due to congenital abnormalities and subsequent failure to thrive. An additional 25 neonatal fawns were excluded from the establishment of reference intervals due to insufficient sample volume or excessive blood sample clotting.

At ∼3–4 months of age, a subset of these same fawns was then resampled as they were weaned from their does in September 2016 (cohort 1) and 2017 (cohort 2). Some animals were not resampled due to being released from the study pens onto the surrounding private preserve and/or were not included in routine handling during subsequent sampling efforts (*n* = 42), or perished due to acute infection before resampling efforts (*n* = 21). As weanlings were handled as part of routine husbandry practices, their health status was evaluated and venipuncture was performed. Deer were categorized as ‘clinically normal’ as per the criteria described above for neonates. Deer were considered to have respiratory disease if they displayed clinical signs including purulent nasal discharge or hacking/rattling cough and accompanying lethargy. Weanlings were also considered ‘sick’ if they tested positive for orbivirus vRNA using molecular methods, even in the absence of clinical disease, and consequently were excluded from the clinically normal group. Therefore, three categories of health status were considered in weanlings: ‘clinically normal’, ‘respiratory disease’, and ‘orbivirus-infected’.

### Sample collection and processing

Samples from neonatal fawns were collected within 24–48 h of birth during the fawning seasons of May-June 2016 and 2017. Immediately following capture, they were momentarily mechanically restrained either by hand or by being placed in a small canvas bag for venipuncture.

Samples were collected from weaned fawns at ∼3–4 months (average 100 days, range 75–126 days) of age. Weanlings were momentarily mechanically restrained using a drop-chute (Mama Deerhandler™, Delclayna Whitetail & Bison Co., Little Falls, MN, USA). Deer were not chemically immobilized, as such drugs have been shown to affect hematological and biochemical analytes (Mautz et al. 1980; Boesch et al. 2011).

All blood samples were collected using 18 G needles by either saphenous (neonates) or jugular (weanlings) venipuncture immediately (within 1–2 min) following restraint and transferred into 6 mL Vacutainer serum separator tubes (Becton Dickinson, Franklin Lakes, NJ, USA) followed by 1 and 6 mL EDTA-dry coated Vacutainer tubes (Fisher Scientific, Hampton, NH, USA). Serum samples were spun for 15 min at 15,000× *g* within ∼1 h of sample collection. EDTA whole blood samples were carefully inverted immediately after collection to ensure proper mixing with anticoagulant. Well-mixed EDTA whole blood was used to prepare two blood films per sample immediately after blood collection. Whole blood and serum samples were kept at 4 °C for <24–72 h before shipment, with the exclusion of serum from the 2017 neonatal fawns, which was frozen at −80 °C within 24–48 h of collection.

All serum and whole blood samples were shipped on ice packs overnight to the University of Miami Avian and Wildlife Laboratory. Serum biochemical analysis included glucose, blood urea nitrogen (BUN), creatinine, calcium (Ca), phosphorus (P), total protein, and alanine aminotransferase (ALT) using a dry chemistry analyzer (Ortho Vitros 250 analyzer, Ortho Clinical Diagnostics, Rochester, NY, USA). A CBC was performed on whole blood samples using a commercial analyzer (Hemavet 950, Drew Scientific, Miami Lakes, FL, USA) and included the following analytes: white blood cell count (WBC), red blood cell count (RBC), hemoglobin (HGB), hematocrit (HCT), mean cell volume (MCV), mean corpuscular hemoglobin (MCH), and mean corpuscular hemoglobin concentration (MCHC). Samples that were visually clotted or hemolyzed more than 1 on a hemolysis scale of 1–3 were excluded from further analyses (*n* = 11 neonates, *n* = 2 weanlings).

Blood films were manually stained with Wright-Giemsa and evaluated with a compound light microscope for overall blood cell distribution (i.e. presence of cellular monolayer, presence of platelet clumps, identification of hemoparasites, etc.), blood cell morphology, and WBC differential. The automated WBC count was used to calculate absolute numbers of the WBC differential, including segmented absolute neutrophils (Absolute Segs ×10^9^/L), absolute lymphocytes (Absolute Lymph ×10^9^/L), absolute monocytes (Absolute Mono ×10^9^/L), absolute eosinophils (Absolute Eos ×10^9^/L), and absolute basophils (Absolute Basos ×10^9^/L). Quantitative platelet evaluation by hematology analyzer and blood film review was not considered accurate due to excessive platelet clumping, and therefore platelet quantification was excluded from the analysis.

Lastly, EDTA-whole blood from all animals was tested for epizootic hemorrhagic disease virus (EHDV) and bluetongue virus (BTV) vRNA by RT-qPCR using the assay as previously described by Wernike et al. (2015).

### Statistical analysis

Development of reference intervals for each blood analyte was conducted using the Reference Interval Advisor freeware v 2.1 (http://www.biostat.envt.fr/reference-value-advisor/) (Geffré et al. 2011). This package develops reference intervals in accordance with the guidelines set forth by the American Society for Veterinary Clinical Pathology (ASVCP) (Friedrichs et al. 2012) in Microsoft Excel. All reference intervals were calculated using this method with 90% confidence intervals (CI). Data were evaluated for normality using the Shapiro-Wilk test (Shapiro and Wilk 1965) and outliers were removed. For variables that were normally distributed, parametric methods were utilized to determine reference intervals. As outlined in Friedrichs et al. (2012), non-Gaussian analytes were either Box-Cox transformed or reference intervals were constructed by non-parametric methodology. For non-parametrically developed reference intervals, 90% CI was calculated using a bootstrap methodology (Geffré et al. 2011).

Further comparative statistical analyses for blood analytes of neonates and weanlings, males and females, and clinically normal and sick weanlings were carried out using the base ‘stats’ package in the statistical program, R v.3.5.0 (R Core Team 2017). Linear mixed effects models (LMMs) were used to assess the fixed effects of age, sex, and health status on the 21 blood analytes using the *lme4* package (Bates et al. 2015). Select analytes were log-transformed to better meet the assumption of normality. For each analyte, a global model was constructed that included age, sex, and health status as fixed effects. To address non-independence of data due to repeated measures of the same individual and batch effects, the year of sampling, the pen the animal was kept in, and individual subject ID were also included in the model as random effects [lmer model: Dependent Variable ∼ Age + Sex + Health Status + (1| Year) + (1| Pen) + (1| SampleID)]. The ‘Anova’ function of the *Car* package was used to calculate the *χ*^2^ and *p*-values and conditional *R*^2^ values were computed using the ‘R.squaredGLMM’ function of the *MuMIn* package (Barton 2015). The residual plots were visually inspected and only models that did not obviously violate the assumptions of linear models were retained (*n* = 13).

To further investigate the differences in blood analytes between males and females of different age groups, a *t*-test with Welch correction was utilized for parametric data while a Mann-Whitney test was performed for nonparametric data. Because the health status of weanlings had three categories (i.e. clinically normal, respiratory disease, or with orbivirus infection), a one-way analysis of variance (ANOVA) was conducted for parametric data, while a Kruskal-Wallis comparison was utilized for nonparametric variables (Kruskal and Wallis 1952). Tukey’s honest significant difference test (Tukey 1949) was conducted on ANOVA results while Dunn’s *post-hoc* test (Dunn 1964) was used for Kruskal-Wallis to further elucidate which health status was causing significant differences.

## Results

### Reference intervals: neonates and weanlings

Reference intervals for clinically normal captive-raised neonatal WTD (*n* = 84) hemogram and serum biochemical data are summarized in Tables 1 and 2. Hematological and serum biochemical reference intervals for clinically normal captive-raised weanling WTD (*n* = 28) are summarized in Tables 3 and 4. Hemoparasites were absent in any neonatal blood film. Four weanlings had rare visible *Plasmodium odocoilei* organisms present in their blood films, though these animals were classified as sick with orbiviral infection (*n* = 1) or respiratory disease (*n* = 2), or had a clotted sample (*n* = 1) and were therefore excluded from the reference interval calculations. Published reference intervals from other studies for neonatal free-ranging and captive fawns are summarized in Tables 5 and 6 for comparison to established reference intervals from this study.

**Table 1.** Mean, median, lower, and upper reference intervals for hematological analytes (90% confidence intervals [CI]) of clinically normal captive-raised neonatal white-tailed deer (*Odocoileus virginianus*) in SI units.

Analyte	Descriptive statistics						95% RI				
	Units	*n*	Mean	Median	Min	Max	Lower limit and 90% CI		Upper limit and 90% CI		Distribution
RBC	×10^12^/L	84	8.73	8.47	4.90	12.5	5.77	5.38–6.26	11.5	11.0–12.1	P
HGB	g/dL	84	8.14	8.00	4.00	12.1	4.26	4.00–6.30	11.28	10.80–11.60	NP
HCT	%	84	27.6	27.0	16.0	42.0	16.30	16.0–22.1	36.8	35.0–42.0	NP
MCV	fL	84	31.7	31.0	28.0	41.0	28.1	28.0–29.0	38.9	35.9–41.0	NP
MCH	pg	84	9.4	9.5	7.0	12.0	7.0	7.0–7.0	12.0	11.0–12.0	NP
MCHC	%	84	29.5	30.0	23.0	36.0	24.0	23.0–24.1	33.0	33.0–36.0	NP
WBC	×10^9^/L	84	2.85	2.70	0.50	7.55	0.74	0.58–0.93	6.36	5.60–7.08	NP, T
Absolute neutrophils	×10^9^/L	79	2.08	1.94	0.36	4.78	0.33	ND–0.46	4.64	4.12–5.13	NP, T
Absolute lymphocytes	×10^9^/L	80	0.66	0.53	0.13	2.21	0.18	0.15–0.22	1.61	1.31–1.89	NP, T
Absolute monocytes	×10^9^/L	81	0.02	0.00	0.00	0.12	0.00	0.00–0.00	0.10	0.08–0.12	NP
Absolute eosinophils	×10^9^/L	81	0.03	0.02	0.00	0.16	0.00	0.00–0.00	0.16	0.12–0.16	NP
Absolute basophils	×10^9^/L	81	0.01	0.00	0.00	0.06	0.00	0.00–0.00	0.06	0.04–0.06	NP

RBC: red blood cell count; HGB: hemoglobin; HCT: hematocrit; MCV: mean corpuscular volume; MCH: mean corpuscular hemoglobin; MCHC: mean corpuscular hemoglobin concentration; WBC: white blood cell count; P: parametric; NP: non-parametric.

**Table 2.** Mean, median, lower, and upper reference intervals (90% confidence intervals [CI]) for serum biochemical analytes for clinically normal captive-raised neonatal white-tailed deer (*Odocoileus virginianus*) in SI units.

Analyte	Descriptive statistics						95% RI				
	Units	*n*	Mean	Median	Min	Max	Lower limit and 90% CI		Upper limit and 90% CI		Distribution
Glucose	mmol/L	75	5.66	5.66	2.86	8.68	3.35	2.97–3.76	8.07	7.71–8.48	P
BUN	mmol/L	76	6.71	6.78	2.50	13.6	2.50	2.50–3.18	12.6	10.0–13.6	NP
Creatinine	µmol/L	76	93.7	88.4	53.0	168	53.0	53.0–61.9	168	160–168	NP
BUN: Crea	–	76	0.07	0.07	0.04	0.17	0.04	0.04–0.04	0.15	0.13–0.17	NP, T
Calcium	mmol/L	75	2.41	2.40	2.00	2.80	2.09	2.00–2.19	2.71	2.61–2.80	NP
Phosphorus	mmol/L	73	3.09	3.17	1.91	4.0	2.13	1.94–2.35	3.88	3.76–4.00	NP, T
Ca: P ratio	–	72	0.79	0.77	0.53	1.17	0.57	0.53–0.61	1.14	1.05–1.17	NP
Protein	g/L	75	56.9	55.0	37.0	93.0	37.9	3.70–42.0	77.7	72.0–93.0	NP
ALT	µkat/L	74	0.68	0.66	0.15	1.17	0.41	0.17–0.48	1.04	0.88–1.17	NP

BUN: blood urea nitrogen; Crea: creatinine; Ca: calcium; P: phosphorus; ALT: alanine aminotransferase.

**Table 3.** Mean, median, lower, and upper reference intervals for blood analytes (90% confidence intervals [CI]) for clinically normal captive-raised weanling white-tailed deer (*Odocoileus virginianus*) in SI units.

Analyte	Descriptive statistics						95% RI				
	Units	*n*	Mean	Median	Min	Max	Lower limit and 90% CI		Upper limit and 90% CI		Distribution
RBC	×10^12^/L	28	18.4	19.2	10.5	24.3	10.5	8.56–12.5	26.4	24.3–28.5	P
HGB	g/dL	25	14.7	14.7	10.7	19.2	10.5	9.50–11.7	18.8	17.7–20.0	P
HCT	%	28	53.9	56.5	31.0	69.0	22.5	ND–37.2	71.3	68.0–73.9	NP, T
MCV	fL	28	29.3	29.0	28.0	30.0	–	–	–	–	–
MCH	pg	28	7.5	7.0	6.0	9.0	–	–	–	–	–
MCHC	%	28	25.3	25.0	20	30	20.1	18.9–21.4	30.4	29.0–31.7	P
WBC	×10^9^/L	27	3.46	3.50	1.6	6.0	0.99	0.39–1.62	5.92	5.25–6.57	P
Absolute neutrophils	×10^9^/L	28	1.51	1.15	0.51	3.36	0.45	0.40–0.55	3.90	2.80–4.99	NP, T
Absolute lymphocytes	×10^9^/L	28	1.92	1.83	0.70	3.62	0.56	0.45–0.78	3.92	3.28–4.61	NP, T
Absolute monocytes	×10^9^/L	28	0.02	0.00	0.00	0.08	–	–	–	–	–
Absolute eosinophils	×10^9^/L	28	0.13	0.09	0.00	0.49	0.000	ND–0.01	0.46	0.32–0.68	NP, T
Absolute basophils	×10^9^/L	28	0.004	0.000	0.0	0.04	–	–	–	–	–

RBC: red blood cell count; HGB: hemoglobin; HCT: hematocrit; MCV: mean corpuscular volume; MCH: mean corpuscular hemoglobin; MCHC: mean corpuscular hemoglobin concentration; WBC: white blood cell count; P: parametric; NP: non-parametric; T: Box-Cox transformed data.

**Table 4.** Mean, median, lower, and upper reference intervals (90% confidence intervals [CI]) for serum biochemical analytes for clinically normal captive-raised weanling white-tailed deer (*Odocoileus virginianus*) in SI units.

Analyte	Descriptive statistics						95% RI				
	Units	*n*	Mean	Median	Min	Max	Lower limit and 90% CI		Upper limit and 90% CI		Distribution
Glucose	mmol/L	26	8.64	8.63	5.10	11.65	5.57	4.68–6.48	11.75	10.8–12.6	P
BUN	mmol/L	26	8.28	8.21	5.35	10.4	4.64	3.63–5.85	11.03	10.4–11.5	NP, T
Creatinine	µmol/L	26	104	97.2	79.6	141	69.9	61.5–78.8	138	128–147	P
BUN:Crea	–	25	0.08	0.08	0.06	0.12	0.05	0.05–0.06	0.13	0.11–0.15	NP, T
Calcium	mmol/L	26	2.64	2.60	2.50	2.80	–	–	–	–	–
Phosphorus	mmol/L	26	3.14	3.18	2.10	3.91	2.12	1.86–2.42	4.20	3.95–4.48	P
Ca:P	–	26	0.86	0.83	0.66	1.29	0.65	0.63–0.68	1.27	1.11–1.45	NP, T
Protein	g/L	26	58.3	58.2	53.0	65.0	50.8	48.8–52.5	65.5	63.1–67.3	P
ALT	µkat/L	26	1.33	1.30	0.77	1.79	0.75	0.62–0.90	1.90	1.74–2.04	P

BUN: blood urea nitrogen; Crea: creatinine; Ca: calcium; P: phosphorus; ALT: alanine aminotransferase.

**Table 5.** Hematological reference intervals for neonatal captive-raised white-tailed deer (WTD; *Odocoileus virginianus*) established in our study, as compared to those established for WTD in previous studies.

Analyte	Units	Our study, neonates	Our study, weanlings	Johnson et al. for penned neonate WTD	Seal and Erickson (1969) for juvenile WTD
WBC	×10^9^/L	0.74–6.36	0.99–5.92	–	–
RBC	×10^12^/L	5.77–11.5	10.5–26.4	7.11–8.51	9.6–13.6
HGB	g/dL	4.26–11.28	10.5–18.8	7.6–9.2	10.4–16.3
HCT	%	16.3–36.8	22.5–71.3	29.1–32.7	32.4–48.6
MCV	fL	28.1–38.9	–	37.4–42.6	30.7–39.6
MCH	pg	7.0–12.0	–	11.4–13.4	–
MCHC	%	24.0–33.0	20.1–30.4	23.8–31	31.3–34.9
Absolute neutrophils	×10^9^/L	0.33–4.64	0.45–3.90	–	–
Absolute lymphs	×10^9^/L	0.18–1.61	–	–	–
Absolute monocytes	×10^9^/L	0–0.01	–	–	–
Absolute eosinophils	×10^9^/L	0–0.16	0–0.46	–	–
Absolute basophils	×10^9^/L	0–0.06	–	–	–

RBC: red blood cell count; HGB: hemoglobin; HCT: hematocrit; MCV: mean corpuscular volume; MCH: mean corpuscular hemoglobin; MCHC: mean corpuscular hemoglobin concentration; WBC: white blood cell count; P: parametric; NP: non-parametric; T: Box-Cox transformed data.

**Table 6.** Serum biochemical reference intervals for neonatal captive-raised white-tailed deer (WTD; *Odocoileus virginianus*) were established in this study, as compared to those established for WTD in previous studies.

Analyte	Units	Our study, neonates	Our study, weanlings	Johnson et al. for penned neonate WTD	Smith et al. for WTD fawns
Glucose	mmol/L	3.35–8.07	5.57–11.75	5.66–7.74	1.85–13.2
BUN	mmol/L	2.50–12.6	4.64–11.03	–	5.64–13.32
Creatinine	µmol/L	53.0–168	69.9–138	–	70.7–286
BUN:Crea	–	0.04–0.15	0.05–0.13	–	–
Calcium	mmol/L	2.09–2.71	–	–	2.30–3.03
Phosphorus	mmol/L	2.13–3.88	2.12–4.20	–	2.36–5.98
Protein	g/L	37.9–77.7	50.8–65.5	46–62	58–85
ALT	µkat/L	0.17–1.04	0.75–1.90	–	–

BUN: blood urea nitrogen; Crea: creatinine; Ca: calcium; P: phosphorus; ALT: alanine aminotransferase.

### Linear mixed effects models and age and sex

**Figure 1.**
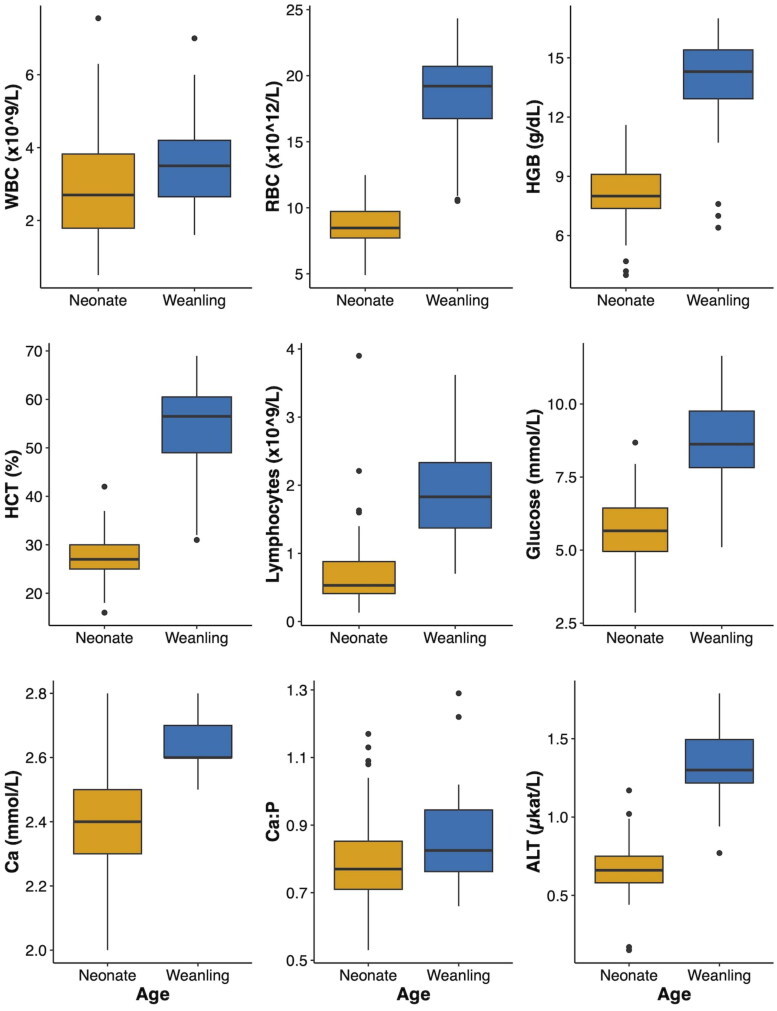
Box plots of all significantly different blood analytes resulting from the comparison between neonatal and weanling white-tailed deer (*Odocoileus virginianus*) fawns by age. WBC: white blood cell count; RBC: red blood cell count; HGB: hemoglobin; HCT: hematocrit; MCV: mean cell volume; MCH: mean corpuscular hemoglobin; MCHC: mean corpuscular hemoglobin concentration; BUN: blood nitrogen urea; Ca: calcium; P: phosphorus; ALT: alanine aminotransferase.

**Figure 2.**
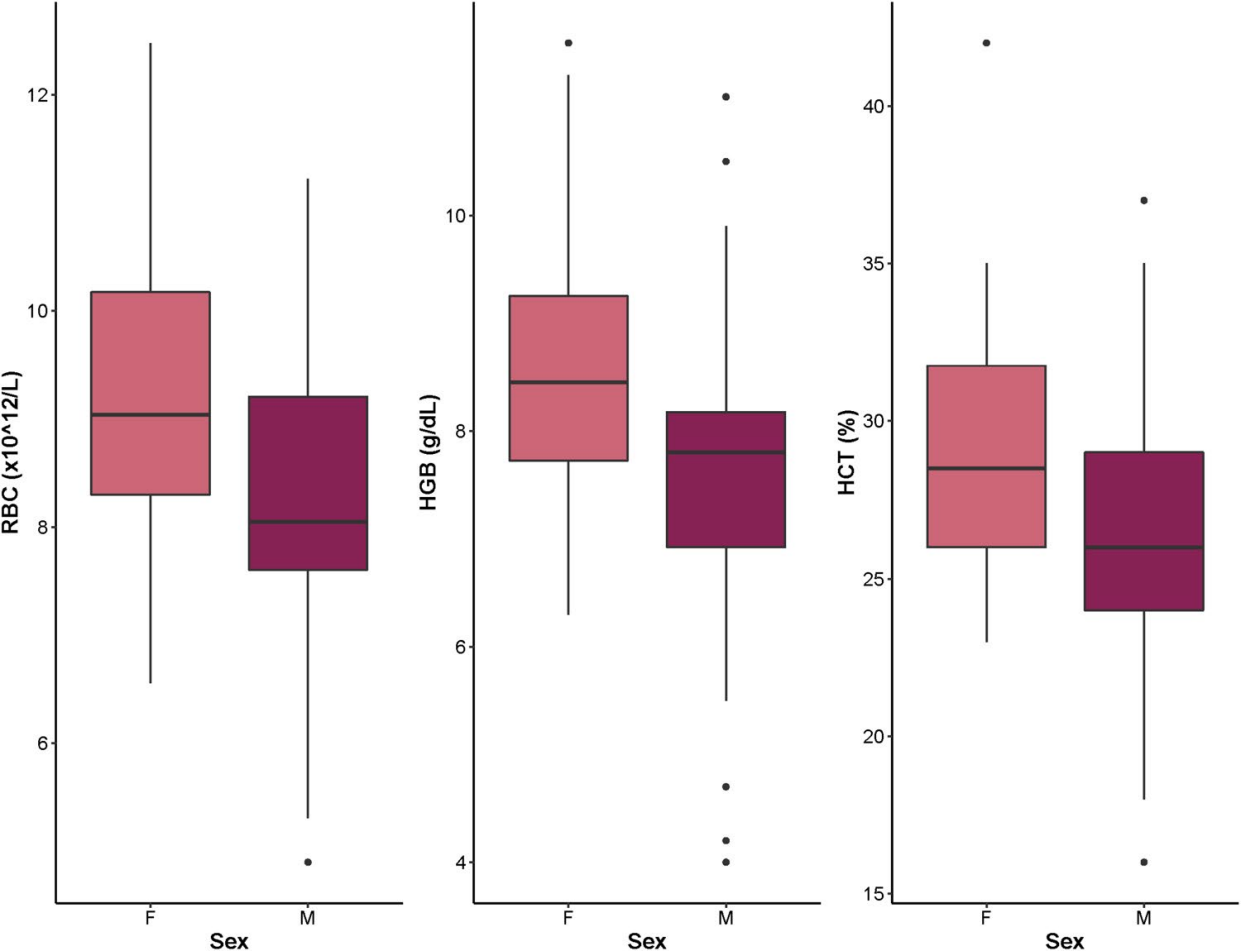
Box plots showing blood analytes that were significantly different in captive-raised female (F) neonatal white-tailed deer (*Odocoileus virginianus*) compared to males (M). RBC: red blood cell count; HGB: hemoglobin; HCT: hematocrit.

Linear mixed models were constructed, and models for 13 analytes met assumptions and were retained. Summary statistics for the fixed effects of age, sex, and health status on these blood analytes are summarized in Table 7. Age had a significant effect on 9/13 (69%) of blood analytes. Weanlings had significantly higher WBC, RBC, HGB, HCT, Absolute Lymphs, glucose, BUN, Ca, and ALT than neonates (*p* < 0.05) (Table 7; Figure 1). Age did not have a significant effect on Creatinine, BUN:Creatinine, P, or Total Protein (*p* > 0.05) (Table 7). Sex had a significant impact on RBC, HGB, and HCT values (*p* < 0.05) (Table 7; Figure 2). These differences were primarily driven by female WTD neonates. They displayed higher mean RBC (9.21 × 10^12^/L *vs.* 8.33 × 10^12^/L; *p* = 0.0037), HGB (8.62 *vs.* 7.74 g/dL; *p* = 0.0071), and HCT (29.1 *vs.* 26.4%; *p* = 0.020) than neonatal males (Figure 2). There were no other significant differences in hematological or serum biochemical analytes between sexes in neonates or between sexes in weanlings (all *p*-values >0.05). There were no significant differences in blood analytes across any of the health states (*p* > 0.05) (Table 7).

**Table 7.** Summary of chi-squared, degrees of freedom, *p*-values, and conditional *R*^2^ values from linear mixed effects models of blood analytes in white-tailed deer fawns.

	Age	Health status	Sex	*R* ^2^ *c*
WBC	***χ*² = 7.2780, *df* = 1, *p* = 0.00698**	*χ*² = 3.2216, *df* = 2, *p* = 0.19973	*χ*² = 0.3406, *df* = 1, *p* = 0.55951	0.46
RBC	***χ*² = 425.4980, *df* = 1, *p* = <2.2e-16**	*χ*² = 2.3245, *df* = 2, *p* = 0.312779	***χ*² = 7.6249, *df* = 1, *p* = 0.005757**	0.87
HGB	***χ*² = 133.8264, *df* = 1, *p* = <2e-16**	*χ*² = 3.0334, *df* = 2, *p* = 0.21944	***χ*² = 5.9537, *df* = 1, *p* = 0.01469**	0.68
HCT	***χ*² = 328.1243, *df* = 1, *p* = <2.2e-16**	*χ*² = 2.1061, *df* = 2, *p* = 0.348873	***χ*² = 7.5032, *df* = 1, *p* = 0.006159**	0.83
Lymphocytes	***χ*² = 90.1337, *df* = 1, *p* = <2e-16**	*χ*² = 2.8732, *df* = 2, *p* = 0.2377	*χ*² = 1.2294, *df* = 1, *p* = 0.2675	0.56
Glucose	***χ*² = 94.3298, *df* = 1, *p* = <2e-16**	*χ*² = 2.0010, *df* = 2, *p* = 0.3677	*χ*² = 1.1164, *df* = 1, *p* = 0.2907	0.61
Creatinine	*χ*² = 3.3119, *df* = 1, *p* = 0.06878	*χ*² = 1.8296, *df* = 2, *p* = 0.40059	*χ*² = 0.4807, *df* = 1, *p* = 0.48812	0.10
BUN:Creatinine	*χ*² = 2.5632, *df* = 1, *p* = 0.1094	*χ*² = 0.2315, *df* = 2, *p* = 0.8907	*χ*² = 0.0223, *df* = 1, *p* = 0.8814	0.17
Ca	***χ*² = 54.8351, *df* = 1, *p* = 1.311e-13**	*χ*² = 3.3597, *df* = 2, *p* = 0.1864	*χ*² = 0.2509, *df* = 1, *p* = 0.6164	0.43
P	*χ*² = 0.9946, *df* = 1, *p* = 0.3186	*χ*² = 0.3864, *df* = 2, *p* = 0.8243	*χ*² = 0.0000, *df* = 1, *p* = 0.9997	0.07
Ca:P	***χ*² = 4.4522, *df* = 1, *p* = 0.03486**	*χ*² = 0.3026, *df* = 2, *p* = 0.85959	*χ*² = 0.1446, *df* = 1, *p* = 0.70374	0.07
Total Protein	*χ*² = 1.1434, *df* = 1, *p* = 0.2849	*χ*² = 1.9071, *df* = 2, *p* = 0.3854	*χ*² = 0.0260, *df* = 1, *p* = 0.8718	0.17
ALT	***χ*² = 171.6989, *df* = 1, *p* = <2e-16**	*χ*² = 0.9732, *df* = 2, *p* = 0.6147	*χ*² = 0.5797, *df* = 1, *p* = 0.4464	0.69

RBC: red blood cell count; HGB: hemoglobin; HCT: hematocrit; MCV: mean corpuscular volume; MCH: mean corpuscular hemoglobin; MCHC: mean corpuscular hemoglobin concentration; WBC: white blood cell count.

Significant coefficients are indicated in bold.

### Comparison of blood analytes in clinically normal and sick weanlings

Twenty-eight clinically normal and 18 sick WTD weanlings were compared, 12 of which had clinical evidence for respiratory tract disease and 6 of which tested positive for orbiviral vRNA (5 were RT-qPCR positive for EHDV, and 1 was RT-qPCR positive for BTV). Absolute monocyte count was marginally significant by Kruskal-Wallis (*p* = 0.04); however, *post-hoc* analysis by Dunn’s test revealed that pairwise comparison of health groups was not driving these differences (all corrected *p* > 0.05). Additionally, MCHC was significantly different among health categories (*p* = 0.027), with *post-hoc* analyses by Tukey’s test showing that HD-positive animals had significantly higher MCHC. However, the average MCHC for HD-positive animals (28.0) was within the reference range of clinically normal individuals established in this study. Health status did not have a significant effect on any other blood analytes between weanlings with orbivirus infection detected by PCR or respiratory disease and clinically normal weanlings, or between weanlings with orbivirus infection and respiratory disease (*p*-values >0.05).

## Discussion

White-tailed deer farming is an important industry with a need for the development of hematological and serum biochemical reference intervals that consider various extrinsic and intrinsic factors. The results of this study demonstrate that intrinsic factors, such as age and sex can impact blood analytes in farmed WTD fawns. Furthermore, we developed de novo hematological and serum biochemical reference intervals for farmed WTD fawns at two critical life stages.

There was appreciable agreement between the blood analyte data established for farmed neonates in our study and those previously published for penned neonatal WTD (Johnson et al. 1968), with most of those data falling within the ranges established in our study except for HGB and MCH, which we found to be lower (4.3 *vs.* 7.6 g/dL, and 7.0 *vs.* 11.4 pg in our study *vs.* Johnson et al. 1968, respectively). There were also some notable differences in blood analytes when comparing our data to Seal and Erickson (1969), specifically RBC and MCHC. The main consideration for this divergence in hematological data includes methodology differences. Seal and Erickson (1969) used a cell counter to measure RBC and a formula to convert packed cell volume to MCHC, and Johnson et al. (1968) measured RBC and WBC *via* hemocytometer and hemoglobin *via* colorimeter. The data in the current study, however, were determined by an automated hematology analyzer that is presumptively more sensitive for the quantification of lower WBC counts. Further considerations for the differences between studies include variations due to diet or environmental factors (e.g. season), as these have been demonstrated to influence hematological analytes (DelGiudice et al. 1992). Additionally, the high density of study animals potentially resulted in the observed higher ranges of WBC counts (e.g. higher density pressure resulting in stress).

There was remarkably close agreement between chemistry reference range data for Texas captive-raised WTD fawns (Smith 2012) and those in our study. However, the lower range for BUN was lower for Florida WTD fawns in our study compared to Texas WTD fawns, suggesting potential dietary differences or analytical differences of chemistry analyzers between studies (e.g. Ortho Vitros 250 using dry chemistry in this study as compared to Roche Modular using wet chemistry analysis in Smith 2012). Creatinine, calcium, phosphorus, and total protein were only marginally lower in our study, indicating that husbandry and possibly geographical location may not strongly influence these analytes. There were only marginal differences between total protein ranges from our study and those previously published by Johnson et al. (1968); however, the lower range for serum glucose in our study was much less, which could be caused by delay in processing due to field conditions, variations in maternal or fawn diet, and/or differences in analytical methods (e.g. the manual Folin-Wu method used for blood glucose estimation by Johnson et al. (1968) is neither considered sensitive nor specific as compared to automated analyzers). Overall, while there is a similarity between our neonatal WTD reference intervals and those previously established for neonatal and adult WTD (Johnson et al. 1968; Powell and DelGiudice 2005; Smith et al. 2012), the differences for various serum biochemical analytes suggest the need for method- and species-specific reference intervals that consider intrinsic factors.

The results of the mixed effects models demonstrate that age and sex are significant factors affecting blood analyte data in WTD. Blood analytes in fawns appear to change rapidly within the first three months after birth, indicating substantial physiological changes associated with growth, including the development of the immune system and changes in metabolism. While White and Cook (White and Cook 1974) reported no significant differences in hematological data with increasing age, this was contradicted by Rawson et al. (1992) who found that RBC, packed cell volume (PCV), and MCHC were all positively correlated with age in WTD fawns and MCV was inversely related. Seal and Erickson (1969) also reported age and sex differences in their blood analyte data. Therefore, age and sex should be considered as important factors when interpreting hematological analytes in WTD fawns.

One limitation of this study was the low number of sick animals in both ‘sick’ fawn groups. There appeared to be no differences between the data for clinically normal weanling WTD and that of orbivirus-infected deer, contradicting previous studies that detailed lymphopenia in response to orbivirus infections (Quist et al. 1997). While our study identified a significant difference in absolute monocytes between clinically normal and sick weanlings, these findings were presumably not biologically relevant, as the diseased animal values were within the established reference intervals and/or the margin of error on the machine and/or the values were close enough that it would not be considered significant by veterinarians. Other studies have found blood analytes indicative of disease and parasite burdens in WTD (Sams et al. 1996; Cray et al. 2019). Our analyses are based on a comparison of blood data from only 29 clinically normal and 18 sick individuals, and we may not have had the sample size to yield statistical significance in blood analyte differences. Additionally, these samples were only collected during one time point in orbivirus infection and respiratory disease presentation and may have been collected too early or late in the course of infection to have been reflected in the blood analyses. Therefore, further studies with larger sample sizes across a time series of infection might be useful to determine the diagnostic performance of blood analytes in diseased WTD fawns.

Despite a sample size compliant with previously established studies for non-companion mammalian species and the use of the recommended ASVCP statistical methodology for reference ranges (Friedrichs et al. 2012), there are a few limitations to this study. Principally, the neonatal fawns originated from a relatively homogenous population that was fed the same diet and came from the same facility, which may limit the utility of extrapolating these ranges to other populations. Despite this, the husbandry practices encountered at the study site were considered representative of the industry standard and therefore may be useful in assessing the health of farmed deer of similar age.

The observed blood analyte data in this study demonstrate the need for future investigations of reference intervals partitioned by age and sex in farmed WTD fawns. The establishment of reference intervals for neonatal and weanling WTD provides a standard for health assessment at critical time points when mortality rates are high primarily due to disease, loss of protective maternal antibodies, and stress of handling (Cook et al. 1971; Vreeland et al. 2004; Harvey 2012). Future studies for the development and application of more sensitive and specific diagnostic biomarkers are warranted to enable rapid identification of diseased WTD for therapeutic intervention and monitoring of WTD populations.

## References

[CIT0001] Anderson DP, Frosch BJ, Outlaw JL. 2007. Economic impact of the United States cervid farming industry. In: Research report 07-4 [Internet]. College Station (TX): Agricultural Food and Policy Center – Texas A&M University [cited 2020 Nov 22]. https://www.afpc.tamu.edu/research/publications/480/rr-2007-04.pdf.

[CIT0002] Barton K. 2015. MuMIn: multi‐Model Inference. R package version 1.15.1.

[CIT0003] Bates D, Mächler M, Bolker B, Walker S. 2015. Fitting linear mixed-effects models using lme4. J Stat Soft. 67(1):1–48. doi: 10.18637/jss.v067.i01.

[CIT0004] Boesch JM, Boulanger JR, Curtis PD, Erb HN, Ludders JW, Kraus MS, Gleed RD. 2011. Biochemical variables in free-ranging white-tailed deer (*Odocoileus virginianus*) after chemical immobilization in clover traps or via ground-darting. J Zoo Wildl Med. 42(1):18–28. doi: 10.1638/2009-0146.1. 22946365

[CIT0005] Brooks JW, Jayarao BM. 2008. Management practices used by white-tailed deer farms in Pennsylvania and herd health problems. J Am Vet Med Assoc. 232(1):98–104. doi: 10.2460/javma.232.1.98. 18167117

[CIT0006] Brooks JW, Kumar A, Narayanan S, Myers S, Brown K, Nagaraja TG, Jayarao BM. 2014. Characterization of *Fusobacterium* isolates from the respiratory tract of white-tailed deer (*Odocoileus virginianus*). J Vet Diagn Invest. 26(2):213–220. doi: 10.1177/1040638714523613. 24590666

[CIT0007] Cauvin A, Dinh ETN, Orange JP, Shuman RM, Blackburn JK, Wisely SM. 2020. Antibodies to epizootic hemorrhagic disease virus (EHDV) in farmed and wild Florida white-tailed deer (*Odocoileus virginianus*). J Wildl Dis. 56(1):208–213. 31298969

[CIT0008] Cook RS, White M, Trainer DO, Glazener WC. 1971. Mortality of young white-tailed deer fawns in south Texas. J Wildl Manage. 35(1):47–56. doi: 10.2307/3799870.

[CIT0009] Cray C, Knibb RI, Knibb JR. 2019. Serum amyloid A and plasma protein electrophoresis fractions in farmed white-tailed deer. J Vet Diagn Invest. 31(3):458–462. doi: 10.1177/1040638719836150. 30852951 PMC6838698

[CIT0010] DelGiudice GD, Mech LD, Kunkel KE, Gese EM, Seal US. 1992. Seasonal patterns of weight, hematology, and serum characteristics of free-ranging female white-tailed deer in Minnesota. Can J Zool. 70(5):974–983. doi: 10.1139/z92-139.

[CIT0011] DelGiudice GD, Mech LD, Seal US, Karns PD. 1987. Effects of winter fasting and refeeding on white-tailed deer blood profiles. J Wildl Manage. 51(4):865–873. doi: 10.2307/3801753.

[CIT0012] Dunn OJ. 1964. Multiple comparisons using rank sums. Technometrics. 6(3):241–252. doi: 10.1080/00401706.1964.10490181.

[CIT0013] Friedrichs KR, Harr KE, Freeman KP, Szladovits B, Walton RM, Barnhart KF, Blanco-Chavez J, American Society for Veterinary Clinical Pathology. 2012. ASVCP reference interval guidelines: determination of de novo reference intervals in veterinary species and other related topics. Vet Clin Pathol. 41(4):441–453. doi: 10.1111/vcp.12006. 23240820

[CIT0014] Gaydos JK, Davidson WR, Elvinger F, Mead DG, Howerth EW, Stallknecht DE. 2002. Innate resistance to epizootic hemorrhagic disease in white-tailed deer. J Wildl Dis. 38(4):713–719. doi: 10.7589/0090-3558-38.4.713. 12528437

[CIT0015] Geffré A, Concordet D, Braun J-P, Trumel C. 2011. Reference value advisor: a new freeware set of macroinstructions to calculate reference intervals with Microsoft Excel. Vet Clin Pathol. 40(1):107–112. doi: 10.1111/j.1939-165X.2011.00287.x. 21366659

[CIT0016] Harvey JW. 2012. Veterinary hematology: a diagnostic guide and color atlas. 1st ed. Amsterdam: Elsevier.

[CIT0017] Hattel AL, Shaw DP, Love BC, Wagner DC, Drake TR, Brooks JW. 2016. A retrospective study of mortality in Pennsylvania captive white-tailed deer (*Odocoileus virginianus*): 2000–2003. J Vet Diagn Invest. 16(6):515–521. doi: 10.1177/104063870401600605. 15586566

[CIT0018] Howerth EW, Stallknecht DE, Kirkland PD. 2000. Bluetongue, epizootic hemorrhagic disease, and other orbivirus‐related diseases. In: Williams ES, Barker IK, editors. Infectious diseases of wild mammals. Ames, IA: Iowa State University Press; p. 77–97.

[CIT0019] Johnson HE, Youatt WG, Fay LD, Harte HD, Ullrey DE. 1968. Hematological values of Michigan white-tailed deer. J Mammal. 49(4):749–754. doi: 10.2307/1378736.

[CIT0020] Kruskal WH, Wallis WA. 1952. Use of ranks in one criterion variance analysis. J Am Stat Assoc. 47(260):583–621. doi: 10.1080/01621459.1952.10483441.

[CIT0021] Mautz WW, Seal US, Boardman CB. 1980. Blood serum analyses of chemically and physically restrained white-tailed deer. J Wildl Manage. 44(2):343–351. doi: 10.2307/3807964.

[CIT0022] Powell MC, DelGiudice GD. 2005. Birth, morphologic, and blood characteristics of free-ranging white-tailed deer neonates. J Wildl Dis. 41(1):171–183. doi: 10.7589/0090-3558-41.1.171. 15827223

[CIT0023] Quist CF, Howerth EW, Stallknecht DE, Brown J, Pisell T, Nettles VF. 1997. Host defense responses associated with experimental hemorrhagic disease in white-tailed deer. J Wildl Dis. 33(3):584–599. doi: 10.7589/0090-3558-33.3.584. 9249705

[CIT0024] R Core Team. 2017. R: A language and environment for statistical computing. Vienna: R Foundation for Statistical Computing. https://www.R-project.org/.

[CIT0025] Rawson RE, DelGiudice GD, Dziuk HE, Mech LD. 1992. Energy metabolism and hematology of white-tailed deer fawns. J Wildl Dis. 28(1):91–94. doi: 10.7589/0090-3558-28.1.91. 1548807

[CIT0026] Ruder MG, Lysyk TJ, Stallknecht DE, Foil LD, Johnson DJ, Chase CC, Dargatz DA, Gibbs EPJ. 2015. Transmission and epidemiology of bluetongue and epizootic hemorrhagic disease in north america: current perspectives, research gaps, and future directions. Vector Borne Zoonotic Dis. 15(6):348–363. doi: 10.1089/vbz.2014.1703. 26086556

[CIT0027] Sams MG, Lochmiller RL, Qualls CW Jr., Leslie DM Jr., Payton ME. 1996. Physiological correlates of neonatal mortality in an overpopulated herd of white-tailed deer. J Mammal. 77(1):179–190. doi: 10.2307/1382719.

[CIT0028] Seal US, Erickson AW. 1969. Hematology, blood chemistry and protein polymorphisms in the white-tailed deer (*Odocoileus virginianus*). Comp Biochem Physiol. 30(4):695–713. doi: 10.1016/0010-406x(69)92149-5. 4187797

[CIT0029] Shapiro SS, Wilk MB. 1965. An analysis of variance test for normality (complete samples). Biometrika. 52(3–4):591–611. doi: 10.2307/2333709.

[CIT0030] Smith ML. 2012. Blood chemistry of free-ranging and captive white-tailed deer (*Odocoileus virginianus*) in Texas [M.Sc. thesis]. Texas A&M University. https://oaktrust.library.tamu.edu/handle/1969.1/ETD-TAMU-2011-05-9143.

[CIT0031] Stallknecht DE, Nettles VF, Rollor EA III, Howerth EW. 1995. Epizootic hemorrhagic disease virus and bluetongue virus serotype distribution in white-tailed deer in Georgia. J Wildl Dis. 31(3):331–338. doi: 10.7589/0090-3558-31.3.331. 8592353

[CIT0032] Tukey JW. 1949. Comparing individual means in the analysis of variance. Biometrics. 5(2):99–114. doi: 10.2307/3001913. 18151955

[CIT0033] Tumbleson ME, Cuneio JD, Murphy DA. 1970. Serum biochemical and hematological parameters of captive white-tailed fawns. Can J Comp Med. 34(1):66–71. 4246006 PMC1319424

[CIT0034] Vreeland JK, Diefenbach DR, Wallingford BD. 2004. Survival rates, mortality causes, and habitats of Pennsylvania white-tailed deer fawns. Wildl Soc Bull. 32(2):542–553. doi: 10.2193/0091-7648(2004)32[542:SRMCAH.2.0.CO;2

[CIT0035] Wernike K, Hoffmann B, Beer M. 2015. Simultaneous detection of five notifiable viral diseases of cattle by single-tube multiplex real-time RT-PCR. J Virol Methods. 217:28–35. doi: 10.1016/j.jviromet.2015.02.023. 25746154

[CIT0036] White M, Cook RS. 1974. Blood characteristics of free-ranging white-tailed deer in southern Texas. J Wildl Dis. 10(1):18–24. doi: 10.7589/0090-3558-10.1.18. 4810210

